# Her Heart's Mistake

**Published:** 1889-08-31

**Authors:** E. D. Cumming

**Affiliations:** Author of "An Ordeal by Silence"


### Her Heart's Mistake.

By E. D. Cumming, Author of "An Ordeal by Silence."

*(Continued from page 332.)*

### Chapter XX.—Warren Goes to Church.

Kate's resolve to adhere to her promise was so consistent in its firmness that all Warren's efforts to move her were fruitless, and at length he ceased his prayers, determining to bide his time until Bairdsley should return. It was impossible to conceive that when the man learned from her own mouth that she did not care for him, he would persist in his demand for her to make good her pledge. But the months which must elapse before he reappeared in Meerut would be a period of torture and suspense to Kate.

A fortnight had passed by since she had received Bairdsley's last letter, and she had neither written to nor heard from him again. His shameless selfishness had destroyed what affection she had ever entertained for him, and her whole being revolted from the thought of becoming his wife; but still she held steadfastly to the belief that the promise she had given was inviolable. She begged Warren to discontinue coming to the house, and when he complied with her request she wrote to him frequently, imploring him to leave the station and abandon her to her fate. But it need hardly be said that he declined to meet her wishes in this respect; he stayed on in his rooms at the club, receiving almost nightly visits from the Doctor, who told him all there might be to tell about her movements and actions.

Dr. Harrison had naturally a deep sense of his indebtedness to Warren. He owed his life to him, and could not therefore treat him as he would have done another petitioner for his daughter's hand. When Bairdsley proposed to marry her, he had listened to all he had to say about pecuniary affairs with the severe impartiality of a judge; and had given his consent because he had no just reason for withholding it. But when he learned Kate's story of her attachment to Warren, and knew that the barrister loved her, he found himself planning and calculating how far his own means might be applied to compensate for the second suitor's poverty. And he arrived at the conclusion that when this unhappy correspondence with Bairdsley came to its natural end, he would signify his intention of allowing his daughter at least two hundred a year, or its equivalent, as soon as she married. His sister's death had placed a considerable sum, which he had set apart for her benefit, at his disposal, and he could not put it to a better use than dower Kate with it. He looked upon Warren as his prospective son-in-law, and they spent many evenings discussing the future; for the barrister had promised to give up the notion of a career in India, and to return to England when the Doctor retired, to resume his practice at home. Meantime, they were waiting for events to develope themselves; but could not hope that matters would change until Bairdsley returned to the plains, or Kate saw her position in a more reasonable light.

The affair was unexpectedly brought to a crisis by a letter from Bairdsley to his *fiancée*. He wrote saying that he had succeeded in persuading his medical advisers that he had sufficiently recovered strength to come back to duty, and that he would cut short his leave and return to Meerut at once. His letter was couched in the old affectionate strain, and the only reference he made to the recent correspondence which had passed between them was to say that he meant to forgive and forget it. The last paragraph contained the whole gist of the epistle. It told Kate that he should expect her to marry him not later than a week after his arrival. She could, he thought, urge no objection to this, in view of their late estrangement, which, for his part, he considered at an end.

Kate read the letter at the breakfast-table under her father's eye, with a face impassive as marble; and she handed it to him in silence without changing colour or moving a muscle of her countenance. Bairdsley's extraordinary egotism had turned her to stone, and she knew no pain; all power to feel had left her.

"You will send no answer to that, Kate," said the Doctor, whose lips were white with rage as he spoke. "You will leave me to deal with this man."

"Father, in heaven's name, don't make it harder for me than it is, or I shall go mad."

Dr. Harrison finished his breakfast without referring to the subject again, but when Kate rose from the table he followed her into her own room, and there, standing before her, spoke his mind calmly, though his blood boiled within his veins as he looked upon her.

"You are deluding yourself with the idea that you are bound in honour to marry Captain Bairdsley. You are no more bound to marry him than you are to marry any man in the lines. You gave a promise, I grant, but you gave it under a misconception that makes it no promise at all. You seem to have forgotten that in keeping this shadow of a pledge you are committing yourself to a perjury that is terrible and real in itself, apart from the miserable consequences. Kate, have you thought that you must stand before the altar and solemnly swear to love, honour, and obey this man? Will it be the truth when you answer 'I will'? You do not love him, and you are not my daughter if you honour him now. You may honestly promise to obey him, but have you ever thought what this vow entails upon you? That you are to be tied for life to a man you care nothing for, and for whom the bond of marriage will soon breed hate."

She made no answer, and the Doctor began again with rising excitement:

"I cannot persuade you of your mistake, but this I tell you, and as your father tell you to bear in mind. I will never take hand or part in your sacrifice. I will never give you to a man whom you cannot honestly say you love. Have you no thought for me? You have none; you deliberately plan for yourself a life of such misery that I had rather kill you with my own hand than see it come to pass. Will your happiness be less to me after your marriage than it is to-day, think you? Can I let my only child pass out of mind as she must pass out of my sight? Will you not realise that you cannot stab me more cruelly than with your own misery?"

Kate raised her eyes to his face with a piteous look of agony that silenced him. Unmanned by it, he turned from her and left the room. He could not say more to her now. He had struck the right chord in making the appeal upon his own behalf. What she would not do for her own or her lover's sake she might do for her father's. The thought moved him deeply, and he went away to his day's work swearing to himself that he would save her yet. Had she seen the grief which he left her room to conceal he might have gained his point that morning. As it was he left the battle half won, and gave his daughter time to persuade herself that not even filial love should make her retract her word.

He found her as firm in her resolve as ever when he returned home that evening, and in utter despair sent over to summon Warren. They renewed the attack together, the Doctor joining in her lover's entreaties that she would marry him by special license, and leave the station at once. They had come to the conclusion that personal fear of Bairdsley's anger had much to do with her obstinacy, and that if she could be brought to understand that she need never see him again, she might give way. For two long hours Warren remained with her, reasoning and pleading with all the earnestness and eloquence he could command. He overcame her at length; she was exhausted with the mental distress in which she had passed the day, and was completely prostrated and unstrung.

"It has come to this, Kate," he said, rising from his seat. "It is my last word. Choose between him and me."

And with a burst of weeping, which frightened him by its unlooked-for violence, she bade him have his way. She had preserved the rigid icy demeanour for so long, that both her father and Warren had almost given up hope. But now, overborne by love and physical weakness, Kate bowed to reason. Warren lost no time, as soon as the point had been gained; he only waited a few minutes to take her in his arms and soothe her, and went straight off to Mr. Wilson, the cantonment chaplain, to make the necessary arrangements with him to marry them on the following morning.

The clergyman's astonishment was great; like everyone else in Meerut he had long been aware that his services would shortly be required to unite Miss Harrison and Captain Bairdsley. And when Warren, muddy and hot after his rapid ride from the corner bungalow, walked into his verandah and summoned him from his bed, it took him ten minutes to comprehend the nature of his errand.

"You have forms of license already signed by the Bishop, have you not?" asked Warren.

"Yes, I've got them; but——"

"But what? It's a sudden business I know, Mr. Wilson; but Dr. Harrison will explain the bearings of the case to-morrow, as, of course, he will come to give his daughter away."

"It's such a very unusual way of doing things, Mr. Warren, that I'm not quite sure whether I'm fully justified in giving the license," said the clergyman, doubtfully. He was a young man who had only lately arrived in the country, and who, to use his own expression, "liked to feel his way."

Warren was not in a mood to bandy words, but he saw that the chaplain had genuine misgivings about the matter, and acknowledged to himself that it must be rather startling to him to be called up on such an affair at midnight. But he set to work to allay doubts with quiet judgment.

"As I say, Mr. Wilson, the Doctor will explain everything to you to-morrow, before you begin the service. If the lady consents and the lady's father consents, and I have no objections to being married, can you suggest any reason for refusing the license?"

"I'm not going to refuse it, Mr. Warren; but really, it is so strange, so unusual; I had almost said irregular."

"Well, that's all right, then. We will be at the church at half-past eight, as we intend to leave by the mid-day train."

Mr. Wilson was not altogether happy about it; it was so very unusual and sudden. But he had no grounds for declining to give the license, and finally undertook to be in waiting at the church, at the hour stated. And having settled this important matter, Warren remounted his pony and galloped home to the club in a state of ecstasy at this unexpected realisation of his dearest hopes.

He was at the telegraph office ten minutes after it opened the next morning, and sent off a message to the P. and O. agents at Bombay, instructing them to reserve a cabin by the homeward bound mail steamer which sailed three days later, for Mr. and Mrs. Warren. His heart glowed as he penned the names, and thought that in two short hours Kate would be his own, beyond the reach of Bairdsley's selfishness or malice. He should know how to deal with the man if he dared to come near her now. He looked at his watch as he emerged from the telegraph bungalow. A quarter to seven. Kate had been up until late last night, and was far from well when he left, so he would not disturb her until it was necessary. He went back to the club and sat down to write a note to her; it was very short, but conveyed all he had to say:

"I arranged everything with Wilson last night, and he will be ready for us at half-past eight. I have wired to engage our passages by the 'Khedive,' sailing on Friday. Am packing up now, that I need not leave you again before we start."

He sent the chit over to the corner bungalow by his bearer, and shutting the doors of his room set to work with feverish haste to make preparations for flight. He had only to pack up his belongings, for Dr. Harrison would settle up his affairs after he left. He huddled away clothing, boots, books, and linen, anyhow, anywhere; picturing Kate busily employed in the same way with her property. What a sudden ending this was to his life in India. He had only been out two months, and here he was about to return home again with a wife. He could hardly believe it, although now he was standing in the midst of bulky portmanteaux strapped and locked, looking round the bare room to see that he had omitted nothing important. There were a dozen small articles scattered about, but he could not delay to collect them. What did they matter? What did he care, though he had to leave everything he possessed. He would go in the clothes he stood up in, and never cast a thought upon the goods and chattels left behind, so long as Kate were at his side.

His attire was not such as civilised custom prescribes for a man to wear at his wedding. He was clothed in a striped flannel shirt, whose collar bulged prominently beyond that of his thin tweed jacket, a pair of white drill trousers, and no waistcoat. He had not had time to shave that morning, and no one who met him cantering out of the compound at twenty minutes past eight would have suspected whither and for what purpose he was bound.

The sun was high in the heavens, and smote scorchingly upon his back as he galloped across the maidan, taking a short cut to the church, with his syce panting after him in the rear. There was a gharry crawling along the road with the windows closed; that must contain Kate and her father, and he would arrive in time to receive them. There was Mr. Wilson waiting at the little vestry door of the ugly yellow building wherein his Sunday duties lay. The bell was silent; the venetians which hid the glass windows were closed. They would be married and safe from ten thousand Bairdsleys in another half-hour, before anyone in Meerut knew it.

He drew rein at the gate of the compound, and walked his pony up the path, wishing the clergyman a pleasant "good-morning" as he came within speaking distance.

"You are punctual," said Mr. Wilson, with a smile, as he dismounted. "Have you got the ring?"

Warren had completely forgotten that necessary, but he always wore an old signet ring, which had belonged to his grandfather. He twisted it off his finger, and, saying that it must answer the purpose, thrust it into his pocket.

They stood at the vestry door, watching the gharry's approach. It drew nearer, while the driver flogged his wretched horse, evidently urged to haste by those within. It arrived at the church gate, but never paused there. Warren felt a strange sinking of the heart as it lumbered past and out of sight. There was no other vehicle on the road. He went into the church and sat down to wait, fidgetting uneasily. A quarter to nine. He went out to see if they were yet visible, but though he strained his eyes to pierce the dancing steam which the heat was drawing from the earth, he could see no sign of his bride. He returned to the church, and tried to pass the time by reading the inscriptions on the mural tablets. Nine o'clock; he went out again, accompanied by Mr. Wilson, but still there was no one to be seen.

"If you will kindly wait here, or return in an hour, we will be ready," he said, unable to curb his anxiety longer. "No doubt something is detaining them, and and I will go over to the bungalow and see."

The awful sense of impending disaster grew stronger and stronger as he urged his pony towards the house at the corner, and culminated in a wild throb of despair as he entered the compound and saw no carriage waiting under the porch.

Dr. Harrison came out to meet him with a telegram in his hand. It was from Bairdsley at Umballa, to Kate:

"Meet me at the station; shall arrive at 1.30 to-day."

*(To be continued.)*

